# Metacognitive Transmission Between Parents and Children in the Context of Anxiety Disorders

**DOI:** 10.1007/s10578-023-01577-y

**Published:** 2023-07-28

**Authors:** Laura Marie Köcher, Silke Schlömer-Böttner, Hanna Christiansen

**Affiliations:** 1 Clinical Child and Adolescent Psychology, Department of Psychology, Philipps-Universität Marburg, Frankfurter Straße 35, 35037 Marburg, Germany; 2 Psychotherapy Practice for Children and Adolescents, Alter Kirchhainer Weg 5, 35039 Marburg, Germany

**Keywords:** Metacognition, Child, Adolescent, Anxiety disorder, Transmission

## Abstract

One in ten children is affected by an anxiety disorder. Current state of research shows that transgenerational transmission as well as positive (POS) and negative (NEG) metacognitive beliefs are relevant in the context of anxiety disorders in youth. We investigated whether transgenerational transmission is also evident in conjunction with POS and NEG and cross-sectionally surveyed POS, NEG, anxiety symptoms and worry in 8-16-year-old children and adolescents with anxiety disorders (*n* = 71) and non-clinical controls (*n* = 40) and one of their parents. Our results revealed significant transgenerational correlations for NEG in both samples, and for POS in the non-clinical sample only. Mediation analysis showed that children’s NEG did at least partly mediate the relationship between parents’ NEG and children’s anxiety and worry. Children’s and parents’ POS did not correlate in either sample with children’s anxiety and worry. Further research on the transgenerational transmission of metacognition and longitudinal data is needed.

## Introduction

In childhood and adolescence, anxiety disorders are the most prevalent of mental disorders, affecting approximately one in ten children or adolescents [1]. Anxiety disorders in childhood and youth have various negative impacts, such as on school performance, sleep, or the social interactions of those affected [2, 3]. In many cases, they are accompanied by comorbid mental disorders such as depression or other anxiety disorders [1, 4]. Anxiety disorders are characterized by certain cognitions [5, 6]: patients with separation anxiety disorders often worry about their parents; those with social phobia often think about potential embarrassment in social situations; and patients with generalized anxiety disorder (GAD) worry excessively about several different life aspects. Such negative cognitions often appear repeatedly and feel difficult to control, which is reflected by the transdiagnostic concept of negative repetitive thinking (NRT). It describes the processes of recurring thoughts regardless of their specific content [7] and is associated with the severity of anxiety symptoms in children and adolescents [8, 9]. Worry appears to be a specifically prominent form of NRT in anxiety disorders [5, 6].

However, it is not the number of worries per se that seems to be characteristic, as individuals with GAD did not differ from non-clinical frequent worriers in their worry number and frequency [10]. What differed was the evaluation of one’s own worries, as patients with GAD reported more negative beliefs about the uncontrollability and danger their worries cause [10]. Such beliefs represent so-called *metacognitions*, that is, thoughts about one’s thoughts. These metacognitive beliefs about worry have been widely studied in children and adolescents over the last two decades and are known to be related to worry and anxiety symptoms (for an overview, see [11]). These relationships are incorporated within the metacognitive model (MCM) of GAD by Wells [12–14] to explain the development and maintenance of excessive worry, anxiety, and anxious behavior. The MCM assumes that an initial thought triggers the use of worry especially when someone holds positive metacognitive beliefs (POS) about worry. POS refer to the assumed benefits of one’s worries, e.g., that they will help getting prepared for future situations. When worry leads to the activation of negative metacognitive beliefs (NEG), i.e., that worry is uncontrollable and dangerous, meta-worry may also occur. Meta-worry is a metacognitive worrying process in which the person’s own worries are the content. When this worry about one’s own worries is experienced, it is followed by the physical experience of anxiety, which reinforces worry. The person then tries to cope with these meta-worries, e.g., by avoiding or trying to suppress unpleasant thoughts. However, since these maladaptive strategies do not help in the long term, meta-worry and perceived anxiety and worry also intensify, thus reinforcing NEG. POS are not necessarily considered to be problematic, but NEG are ascribed to be specifically relevant for the development and maintenance of anxiety disorders [14]. This is supported by descriptively larger correlations between anxiety and worry with NEG [i.e., 11] and significant differences in NEG, but not POS when comparing children and adolescents with anxiety disorders to non-clinical controls [15, 16].

Although the MCM generally provides substantial evidence in childhood and youth [i.e., 11], the development, maintenance and change in metacognitive beliefs have seldom been studied. Studies on the transgenerational transmission of anxiety disorders show that a child’s risk of having any anxiety disorder is about four-to-five-times higher when its parents have an anxiety diagnosis [17, 18]. A parent’s symptoms of a particular anxiety disorder also approximately double the risk of a child for developing the same symptoms [19]. Especially anxious, controlling, and overprotective parental behavior is cross-sectionally and longitudinally associated with children’s anxiety [20–22]. Moreover, mothers of children and adolescents with anxiety disorders have been shown to be more protective and critical [23]. In turn, overreactive parenting is positively associated with the severity of parenting stress [24]. This particular form of stress concerns specifically the stress parents experience associated with being a parent and the effects of having a child on relationships or one’s (mental) health [24] and cross-sectional and longitudinal associations have been demonstrated coinciding with the child’s internalizing symptomatology in childhood and adolescence [24–26]. Parenting stress of mothers also correlates with children’s anxiety symptoms [27]. Looking specifically at the cognitive components of anxiety disorders, parental overprotection, rejection, and too little warmth are also associated with children’s worry [28]. Research by Donovan et al. [29, 30] also showed a direct positive association between the worry of parents and children, and a higher level of worry in parents of children with anxiety disorders. A closer look reveals a mediating role of children’s NEG in this parent-child-worry-association [30]. Lønfeldt et al. [31] reported small-to-moderate correlations between children’s POS and their mother’s anxiety and stress symptoms. In addition, Lønfeldt et al. [32] also observed correlations between controlling parental behavior experienced by the child and children’s POS and NEG. Metacognitions also mediated the relationship between perceived maternal control and children’s anxiety. Gallagher and Cartwright-Hatton [22] also partially showed such a mediation in the association between adolescent’s perceived overreactive parental discipline and their anxiety. Metacognitions thus appear to be relevant for the transgenerational transmission process of anxiety disorders. So far, few studies have also investigated specifically the metacognitive transmission effects of parents on their children, and preliminary findings suggest that children’s POS and NEG are related to their parents’ corresponding metacognitions: mothers’ and children’s POS and NEG correlated in a study by Esbjørn et al. [33], and metacognitions of mothers and children correlated with children’s anxiety and worry. Children’s metacognitions fully mediated the relationship between maternal metacognitions and children’s anxiety, and partially mediated the relationship between maternal metacognitions and children’s worry.

Based on these findings, it seems plausible to assume that parents’ metacognitive beliefs might be involved in the development of their children’s POS and NEG, which in turn could promote the development of excessive worry and anxiety disorders. On the other hand, two other studies did not detect such transgenerational associations in conjunction with either POS or NEG [30, 34]. So far, all these studies examined non-clinical samples. To our knowledge, only one compared POS and NEG of parents of children with anxiety disorders and parents of healthy controls, but did not identify differences in POS or NEG [29]. The aim of the present study is thus to investigate further these metacognitive transmission processes between parents and their children. We aim to replicate the results of Esbjørn et al. [33], specifically for POS and NEG. Therefore, we are also assuming that parents’ metacognitions are related to their children’s anxiety and worry, and that these relationships are mediated at least partially by children’s metacognitions. Because of previously ambiguous results on the role of POS [i.e., 11], we examined them here in an exploratory manner. Moreover, we want to investigate whether these mediation models are applicable in a clinical sample by looking at children and adolescents with diagnosed anxiety disorders and their parents. We were also interested in the roles of parent’s anxiety symptoms, worry, and parenting stress, all of which are relevant for anxiety transmission, but about which we know very little so far in the context of metacognitive beliefs. Significant associations have been reported between mothers’ anxiety symptoms with children’s POS and NEG [31] and of parents’ worry with children’s NEG [30]. As, to our knowledge, parenting stress has not yet been studied at all in this area, we investigated the associations between children’s metacognitive beliefs with parents’ anxiety symptoms, worry, and parenting stress exploratively.

## Method

In this study, we report findings based on data from the „My Worries-Your Worries“-study surveyed between 2015 and 2020. Philipps University Marburg’s Ethics Commission approved this study under the reference number 2014-47k (2 September 2014) and 2016‐34k (9 November 2016). This study only reports data for matched parent-child-dyads recruited since 2016, as parents were first assessed in the study from that time onward. Data from the entire „My Worries-Your Worries“-child/adolescent-sample will be published elsewhere. Overall, the present sample consisted of *N* = 111 parent-child-dyads, thereof *n* = 71 children and adolescents with anxiety disorders and *n* = 40 children from the non-clinical school sample.

### Participants and Procedure

For the clinical sample, children and adolescents were recruited at the Child and Adolescent Psychotherapy Outpatient Clinic of the Philipps-University of Marburg (*n* = 13) and outpatient, day-patient and inpatient clinics of a psychiatric hospital for children and adolescents located in Wetzlar and Herborn (*n* = 55). Families contacted the clinics themselves for treatment and were invited to participate in the study if the child was 8–16 years of age and diagnosed with an anxiety disorder according to the International Classification of Diseases, 10th edition (ICD-10 [35]). Families were included in the study if the children’s diagnostic status was confirmed in the family’s report in the questionnaires for anxiety- and obsessive-compulsive-disorders (ANZ) of the diagnosticsystem of mental disorders according to ICD-10 and DSM-IV for children and adolescents (DISYPS-II [36]). For children under 11 years of age, diagnostic status was assessed on the basis of parental judgment, since ANZ norms are not available for self-report at that age. For older children, we assigned a diagnosis if the values of either the parent’s or the child’s judgment fell within the clinically elevated range when compared to the norm sample (cut-off: Stanine score ≥ 8). For the non-clinical convenience sample, children and adolescents were recruited from different schools and sports clubs in Baden-Württemberg, Bavaria, Hesse, Hamburg, Lower Saxony and North Rhine-Westphalia. They were invited to participate if they were 8–16 years of age. After families expressed their interest in the study, they were informed verbally and in writing about the study, and parents gave written informed consent for inclusion. All participating children also consented verbally to participating as well as in writing if they were 12 years of age or older. As a reward for participating, families could decide to take part in a raffle to win one of ten cinema vouchers. Paper-pencil questionnaire packages were filled out by the families at home or during the clinic stay and returned to the research team directly or by mail.

### Child Measures

#### Demographic Variables and Socioeconomic Status

Children’s age, gender, and school type were surveyed via a short questionnaire. Children and adolescents also filled out a modified version of the *Family Affluence Scale II* (FAS-II [37]) assessing their families’ socio-economic status. They answered four items (own room, family car, family computer, vacation last year) on a simplified dichotomous *Yes*/*No*-answer format. Yes-answers where summated. Parents’ age, sex and specification of the primary caregiver of the child were assessed as part of the Parenting Stress questionnaire, which is described below.

*Questionnaires for anxiety- and obsessive-compulsive-disorders (ANZ) of the DISYPS-II* [36]. Anxiety symptoms of children and adolescents were assessed via the self- and parent-report-versions of ANZ. Children/Adolescents and parents rated 33 Items on a four-point Likert scale (0 = *not at all* to 3 = *especially*). Both versions consist of subscales for separation anxiety, GAD, social phobia and specific phobia based on DSM-IV. A total scale includes the subscales’ 31 items as well as two items measuring obsessive-compulsive-symptoms. The questionnaire was validated and normed across a large sample of children aged 11 to 18 years (self-report-version) and 4 to 18 years (parent-report-version), respectively. The total scale reached sufficient values for the self- and parent-report-versions’ internal consistency [36]. Cronbach’s alpha in our clinical sample was α_pooled_ = 0.860 for the self-report version and α_pooled_ = 0.876 for the parent-report-version, and for the non-clinical sample α_pooled_ = 0.889 and α_pooled_ = 0.889 respectively.

*Perseverative thinking questionnaire* (PTQ, [38]). As a transdiagnostic measure of children’s NRT, we used the PTQ. The questionnaire is based on 15 items (i.e., “I think of many problems without solving any of them”), which are answered on a five-point Likert scale (0 = *never* to 4 = *almost all the time*). This measure has adequate psychometric qualities for adults and high internal consistency in a sample of 11- to 19-year-olds [38, 39]. Our samples achieved comparable internal consistency (clinical sample: α_pooled_ = 0.949, non-clinical sample: α_pooled_ = 0.921).

*Measure of excessive worry content – child version* (EWC-C [40]). To measure the content of excessive worries, we adapted the approach from Bacow et al. [41, 42] for the German language. The authors developed items to measure the severity of the worry domains described in the Anxiety Disorders Interview Schedule, Child Version [43] on a nine-point Likert scale. For the German version, we used the worry domains from the GAD-section of the Structured Diagnostic Interview for Mental Disorders in Children [44] as described by Piepenbreier [40]. The severity of the resulting worry domains (family, school/sport achievement, everyday events, friends/social interactions, health/self, health/others, current events like accidents or natural disasters) was measured with one item each on a simplified five-point Likert-scale (0 = *no worries at all*; 4 = *very severe worries*). An additional open-ended item was included to give participants the opportunity to name additional worry domains. Compared to other worry-questionnaires like the Penn State Worry Questionnaire for Children (PSWQ) [45], the advantage of this method is that it allows both worries’ frequency to be measured, as well as their content. In the present sample, Cronbach’s alpha was α_pooled_ = 0.789 for the clinical and α_pooled_ = 0.851 for the non-clinical sample.

*German Metacognitions Questionnaire for children* (MKF-K [46]). Children’s metacognitive beliefs were measured with the MKF-K, which relies on the German translation of the Metacognition Questionnaire-30-item-version [47]. This questionnaire includes 30 items (i.e., “When I worry, I can think more clearly”) on a four-point Likert scale (0 = *not at all* to 3 = *completely)*. Factor analysis resulted in four of the corresponding five subscales from the adult version (POS, NEG, cognitive confidence, cognitive self-consciousness) with adequate reliability and validity [46]. We used the POS and NEG subscales in our study. For the clinical sample, Cronbach’s alpha resulted in α_pooled_ = 0.802 for POS and α_pooled_ = 0.741 for NEG. In the non-clinical sample, Cronbach’s alpha was α_pooled_ = 0.894 for POS and α_pooled_ = 0.568 for NEG.

### Parent Measures

*German Brief Symptom Inventory* (BSI [48, 49]). We employed the BSI to screen parents’ anxiety symptoms and overall mental burden. This inventory contains 53 items assessing symptoms during the last seven days. The answer-format is a five-point Likert scale (0 = *not at all* to 4 = *very strong*). In addition to the total Global Severity Index, nine subscales can be formed (somatization, compulsiveness, uncertainty in social contact, depression, anxiousness, aggressiveness, phobic fear, paranoid thinking and psychoticism). For our analysis, we added up the values from the anxiousness and phobia symptom subscales to measure anxiety symptoms. Adequate reliability and validity for the BSI has been demonstrated [50]. For the clinical sample’s parents, Cronbach’s alpha was α_pooled_ = 0.834 for the anxiety-subscale, α_pooled_ = 0.839 for the phobic-fear-subscale. In the non-clinical sample, internal consistency was α_pooled_ = 0.716 and α_pooled_ = 0.653, respectively.

*Measure of excessive worry content – adult version* (EWC-A [51]). To measure parents’ excessive worry content, we applied the procedure described for the EWC-C to adults. For this purpose, we selected questions from the GAD section of the adult version of the Diagnostic Interview for Mental Disorders [52] resulting in the worry domains family, friends/social interactions, health/self, health/others, current events (like war, accidents or natural disasters). In accordance with the children’s version, these five items were answered on a five-point Likert scale (0 = *no worry at all* to 4 = *very severe worry*) and an additional open-ended item for other worry was included. For the clinical sample’s parents, Cronbach’s alpha was α_pooled_ = 0.832 and α_pooled_ = 0.849 for the parents of the non-clinical sample.

*German Short Form of the Metacognitions Questionnaire* (MKF-30 [53]). To assess parent’s metacognitions, we applied the German MKF-30. This measure consists of 30 items (i.e., “If I worry, I avoid problems in the future”) on a four-point Likert scale (0 = *do not agree* to 3 = *strongly agree*). MKF-30 contains subscales for POS, NEG, cognitive confidence, cognitive self-consciousness and beliefs about needing to control thoughts as well as a total scale. The original five-factor-structure and psychometric properties were also validated for the German version [53, 54]. We used POS and NEG subscales to measure the metacognitions of interest in parents. Internal consistency in the parents of the clinical sample was α_pooled_ = 0.634 for POS and α_pooled_ = 0.812 for NEG and α_pooled_ = 0.802 and α_pooled_ = 0.503 for parents of the non-clinical sample, respectively.

*Parenting stress questionnaire* (ESF [55]). The ESF measures parenting-related stress factors on a total scale and four subscales (parenting stress, role restriction, social support, partnership) with 38 items to be answered on a four-point Likert scale (0 = *does not apply*, 3 = *does apply exactly*). Normed and validated pre-school-and school-age-versions are available. In our study, we used the school-age-version and the overall scale. Adequate internal consistency values have been reported [55]. In our study, Cronbach’s alpha for the total scale was α_pooled_ = 0.773 for parents of the clinical sample and α_pooled_ = 0.880 for parents in the non-clinical sample.

### Statistical Analyses

We used IBM SPSS Statistics 27 for statistical analyses. First, we carried out preliminary analyses, checked for missing data, exanimated patterns of missing values and ran Little’s MCAR test. In case of missing values completely at random (MCAR), we than performed multiple imputation. Second, we ran two-tailed *t*-tests to test for mean differences between the samples with Bonferroni correction as well as Welch correction if applicable. Subsample-assignment relationships with the categorial variables were tested with Pearson-χ^2^-tests. Third, we calculated Pearson’s product-moment correlations of outcome variables. We had no specific hypothesis regarding relationships with children’s sex and age, and used two-tailed testing; all other relationships were tested one-tailed. Potential group differenced in correlations were tested via Fisher’s *z*-transformation and back-transformed confidence limits according to Zou [56]. Fourth, we tested our postulated mediation of the relationship between children’s metacognitive beliefs and their anxiety symptoms/worry by corresponding parents’ metacognitive beliefs according to Hayes’ [57] description with the PROCESS 4.1 macro [58]. As recommended by Hayes [57, 59], we calculated confidence intervals using 10,000 bootstrap samples and set a starting value for the pseudorandom number generator for each calculation. If mediation analysis was not possible, we conducted two-tailed multiple regression analysis on children’s metacognitive beliefs as outcome variables. To interpret effect sizes, we followed the Conventions of Cohen [60] for *d* (0.8 = large, 0.5 = medium, 0.3 = small) and for *r*, Cramer’s *V* and φ (0.5 = large, 0.3 = medium, 0.1 = small).

## Results

### Missing Data

Overall, 104 study variables on the item level had missing data in more than 5% of cases. At the item level, 94.2% of the total values were available, whereas 5.8% of the total values missing. Most cases of missing data concerned the complete items of BSI (11 cases) and ESF (6 cases). Examination of the patterns of missing values revealed moreover that the optional item in EWC-A was missing in 21%, the optional item of the EWC-C missing in 16%, and several items of BSI were missing in 9%. Other patterns of missing values were present in less than 5% of cases. Little’s MCAR test yielded a non-significant result (χ^2^(16,907) = 16664.784, *p* = .907), speaking for MCAR. We performed multiple imputation of missing values with *m* = 20 imputed data sets separately for the clinical and the non-clinical samples (compare [61]). In the imputation model, we included variables relevant to the research questions (items from ANZ self-report-version, ANZ parent-report-version, BSI, PTQ, EWC-C, EWC-A, MKF-30, MKF-K, ESF) as well as a set of auxiliary variables as predictors only (FAS, children’s and parents’ age and sex; and recruitment site for clinical sample). Minimum and maximum values were defined for each item to prevent estimation of out-of-range values. Scale values and analyses conducted for hypothesis testing were then formed separately for each imputed data set and pooled afterwards. If available, we applied the pooling method in SPSS. Otherwise, pooling was done manually applying Rubin’s [62] rules of integration. When bootstrapping, we applied the MI boot method [63] to pool confidence intervals which yielded good results in their simulation study.

### Sample Description

In both samples, there were five outliers and no extreme values. Since there were no indications of response patterns, inadmissible responses or implausible responses in any of the cases, we therefore included those data for further analyses. Table 1 displays information about demographic variables in the clinical and the non-clinical samples. This information is derived from our original data set, as demographic variables were only included as predictors in the imputation model. Of the clinical sample, *n* = 3 families were excluded because no ANZ-diagnosis had been fulfilled according to the aforementioned criteria. The final sample for data analysis was *N* = 108, thereof *n* = 68 (62.96%) in the clinical sample. In the clinical sample, clinically relevant symptoms of separation anxiety disorder were present in *n* = 49 (72.06%), GAD in *n* = 47 (69.12%), social anxiety disorder in *n* = 46 (67.65%), and specific phobia in *n* = 57 (83.82%).

To compare variables between the clinical and non-clinical subsample, the Kolmogorov-Smirnoff-test results and visual inspection suggested normal distribution had been violated for children’s age, parents’ age and family wealth, parents’ anxiety/phobia symptoms, parents’ POS and NEG, children’s POS and NEG in at least one subsample. However, since the *t*-test is considered robust to a violation of the normal distribution assumption above appropriate group sizes, we still ran testing as planned. The Levine-test was significant for children’s age, family wealth, children’s NEG, parents’ NEG and parent-reported child anxiety, so we assumed heteroskedasticity and applied Welch-correction. There was a significant relationship between sample and school type and primary caregiver (compare Table 1), but no significant relationship between sample assignment and children’s or parents’ sex. The clinical sample reported lower family wealth as well as higher scores on children’s anxiety symptoms children’s NEG, children’s NRT, parents’ anxiety/phobic fear and parenting stress.

### Correlations of Children’s Metacognitive Beliefs

Intercorrelations between outcome measures are demonstrated in Table 2. In the clinical sample, POS were associated with child-reported anxiety, parent-reported child anxiety and parents’ worry. NEG was positively associated with children’s age, child-reported anxiety, parent-reported child anxiety, children’s worry, children’s NRT, parents’ anxiety/phobia symptoms, parents’ POS and parents’ NEG. Neither POS nor NEG correlated with parenting stress. POS and NEG did not correlate with each other. POS in the non-clinical sample were associated with children’s NRT, parents’ POS and NEG. NEG were positively associated with child-reported anxiety, parent-reported child anxiety, children’s worry, children’s NRT, parent’s NEG. Neither POS nor NEG correlated with parents’ anxiety/phobia symptoms, parents’ worry or parenting stress. POS and NEG again did not intercorrelate significantly. When comparing the reported correlations of interest, only the correlation of POS in parents and children differed between samples, being higher in the non-clinical sample (*r*_clinical_-*r*_non−clinical_ = − 0.363, 95%-CI [0.097; 0.604]).

**Table 1:** Descriptive statistics for clinical and non-clinical samples

Participating child/adolescent	Clinical (*n* = 68)	Non-clinical (*n* = 40)	Teststatistic
Age, *M* (*SD*), Range	11.89 (2.43), 8–16	11.54 (1.54), 9–15	*t*(105,358) = 0.903
Sex, *n* (%) female	42 (61.8)	19 (47.5)	χ^2^(1) = 2.085
School type^a^, *n* (*%*)			χ^2^(6) = 32.998^***^, *V* = 0.57
Elementary school	18 (26.5)	10 (25.0)	
Grammar school	11 (16.2)	24 (60.0)	
Compr. secondary school	24 (35.3)	1 (2.5)	
Secondary school	6 (8.8)	3 (7.5)	
School for special needs	5 (7.4)	-	
FAS-II^a^,	2.32 (1.19)	3.72 (0.51)	*t*(96.091) = -8.268^***^, *d* = -1.39
ANZ_child,_*M* (*SD*)	36.63 (14.61)	19.84 (12.18)	*t*(1,853,094) = 6.120^***^, *d*_pooled_ = 1.22
ANZ_parent_, *M* (*SD*)	28.79 (15.17)	9.66 (8.85)	*t*(9,083,225) = 8.282^***^, *d*_pooled_ = 1.45
MKF-K POS, *M* (*SD*)	2.89 (2.90)	2.45 (3.02)	*t*(91,485) = 0.750
MKF-K NEG, *M* (*SD*)	5.87 (3.61)	3.48 (3.61)	*t*(45,901) = 4.133^***^, *d*_pooled_ = 0.75
EWC-C, *M* (*SD*)	16.43 (7.03)	14.04 (6.81)	*t*(63,027) = 1.722
PTQ, *M* (*SD*)	29.43 (14.74)	19.49 (11.38)	*t*(716,235) = 3.666^***^, *d*_pooled_ = 0.73
**Participating parent**	** Clinical** ***(n = 68)***	** Non-clinical** ***(n = 40)***	** Teststatistic**
Age^b^, *M* (*SD*), Range	41.57 (5.25), 31–51	43.05 (6.86), 30–63	*t*(89) = -1.164
Mother, *n* (%)	60 (88.2)	39 (97.5)	χ^2^(1) = 2.830
Farther, *n* (%)	8 (11.8)	1 (2.5)	
Main caregiver^c^			χ^2^(2) = 25.830^***^, *V* = .50
Mother, *n* (%)	55 (80.9)	21 (52.5)	
Farther, *n* (%)	5 (7.4)	-	
Both, *n* (%)	4 (5.9)	19 (47.5)	
BSI-A/P, *M* (*SD*)	8.36 (7.94)	2.74 (3.96)	*t*(44,839) = 4.898^***^, *d*_pooled_ = 0.84
ESF, *M* (*SD*)	54.67 (12.11)	45.84 (16.20)	*t*(104,589) = 3.223^***^, *d*_pooled_ = 0.65
MKF-30 POS, *M* (*SD*)	3.45 (2.81)	2.79 (3.32)	*t*(152,519) = 1.106
MKF-30 NEG, *M* (*SD*)	5.20 (4.07)	4.24 (2.68)	*t*(184,726) = 1.484
EWC_parent_, *M* (*SD*)	11.98 (5.40)	10.12 (5.44)	*t*(23,541) = 1.722

*Notes*. Standard division for imputed data calculated manually based on pooled standard errors estimated in SPSS. FAS−II = Family Affluence Scale II, child = assessment of child/adolescent, parent = assessment of parent, ANZ = Questionnaires for anxiety− and obsessive−compulsive−disorders of DISYPS−II, EWC−C = Measure of excessive worry content – child version, PTQ = Perseverative Thinking Questionnaire, MKF−K = Metacognitions Questionnaire for children, POS = positive beliefs about worry, NEG = negative beliefs about worry, BSI−A/P = Brief Symptom Inventory, sum score of Anxiousness and Phobic fear scales, ESF = Parenting stress questionnaire, EWC−A = Measure of excessive worry content – adult version, MKF−30 = Metacognitions Questionnaire – short version.

^a^_*n* = 102,_^b^_*n* = 91,_^c^_*n* = 104. ***p < .001. *V* = Cramer−*V*, d = Cohen’s d_

### Correlations of Parents’ Metacognitive Beliefs

POS and NEG in the clinical sample correlated significantly with parent-reported child anxiety, children’s NEG, children’s worry, children’s NRT, parents’ anxiety/phobia symptoms and parents’ worry. POS, but not NEG were moreover also associated with children’s age and parenting stress. POS and NEG did intercorrelate significantly here. In the non-clinical sample, POS correlated significantly and negatively with children’s age and parenting stress, while positive correlations appeared for children’s POS, parents’ anxiety/phobia symptoms and parents’ worry. For NEG, we identified correlations with child-reported anxiety, parent-reported child anxiety, children’s POS, children’s NEG, children’s worry, children’s NRT, parents’ anxiety/phobia symptoms, parenting stress and parents’ worry. POS and NEG did not intercorrelate significantly. Only one of the correlations of interest differed between groups: The relationship between POS and NEG was higher in the clinical sample (*r*_clinical_-*r*_non−clinical_ = 0.458, 95%-CI [-0.779; − 0.306]). Intercorrelations are illustrated in Table 2.

### Mediation Analysis for NEG

**Fig. 1:**
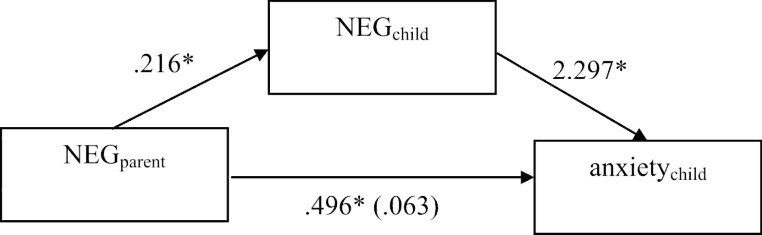
Pooled results of Mediation predicting children’s anxiety (clinical sample); *p < .05

**Fig. 2:**
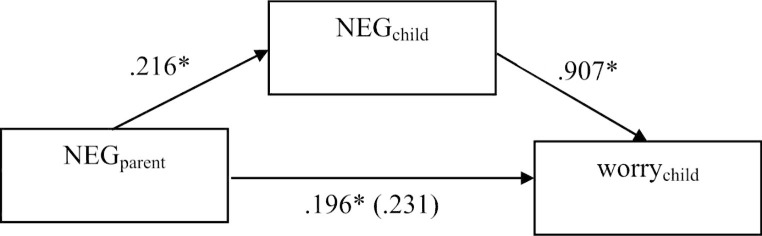
Pooled results of Mediation predicting children’s worry (clinical sample); *p < .05

As children’s NEG correlated significantly with parents’ NEG, children’s anxiety and children’s worry in both samples, we ran mediation analyses as planned. Because of the violation of residual homoskedasticity, significance tests and confidence intervals were based on the robust standard error [64] in both samples. For the clinical sample, the total effect of mediation predicting anxiety was not significant (*B*_pooled_ = 0.560, 95%-CI_pooled_ [-0.211; ∞]). Because of Hayes’ [57] recommendation to test the indirect effect even if the total effect is not significant, we nevertheless conducted the additional analyses to test mediation, which resulted in a significant indirect effect of parents’ NEG on children’s anxiety via children’s NEG (*B*_pooled_ = 0.496, 95%-CI_pooled_ [0.070; ∞]), while the direct effect of parents’ NEG on children’s anxiety was not significant here (*B*_pooled_ = 0.063, 95%-CI_pooled_ [-0.548; ∞]). This speaks for a full mediation of children’s NEG for the relationship of parents’ NEG and children’s anxiety (see Fig. 1). To predict worry, the total effect was significant (*B*_pooled_ = 0.427, 95%-CI_pooled_ [0.058; ∞]). The indirect effect of parents’ NEG on children’s worry via children’s NEG was also significant (*B*_pooled_ = 0.196, 95%-CI_pooled_ [0.020; ∞]), whereas no significant result appeared from the direct effect of parents’ NEG on children’s worry (*B*_pooled_ = 0.231, 95%-CI_pooled_ [-0.114; ∞]). This also speaks for a full mediation of children’s NEG for the relationship of parents’ NEG and children’s worry (see Fig. 2).

**Fig. 3:**
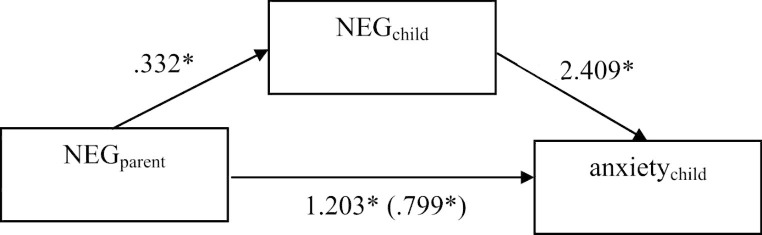
Pooled results of Mediation predicting children’s anxiety (non-clinical sample); * *p* < .05

**Fig. 4:**
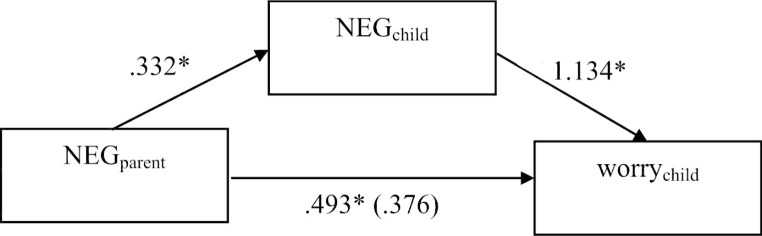
Pooled results of Mediation predicting children’s worry (non-clinical sample); * *p* < .05

In the non-clinical sample, the mediation predicting anxiety showed a significant total effect (*B*_pooled_ = 2.002, 95%-CI_pooled_ [0.966; ∞]). Both the direct effect of parents’ NEG on children’s anxiety (*B*_pooled_ = 1.203, 95%-CI_pooled_ [0.116; ∞]) and its indirect effect on children’s anxiety via children’s NEG (*B*_pooled_ = 0.799, 95%-CI_pooled_ [0.008; ∞]) was also secured against zero, showing partial mediation of children’s NEG for the relationship of parents’ NEG and children’s anxiety (see Fig. 3). The mediation predicting worry also yielded a significant total effect (*B*_pooled_ = 0.869, 95%-CI_pooled_ [0.139; ∞]). While the indirect effect of parents’ NEG on children’s worry via children’s NEG was significant (*B*_pooled_ = 0.493, 95%-CI_pooled_ [0.020; ∞]), its direct effect on children’s worry yielded a non-significant result (*B*_pooled_ = 0.376, 95%-CI_pooled_ [-0.245; ∞]). This suggests a full mediation of children’s NEG for the relationship of parents’ NEG and children’s worry (see Fig. 4).

### Mediation Analysis for POS

As no correlation between children’s and parents’ POS was revealed in the clinical sample, we did not conduct mediation or regression analysis here. Children’s and parents’ POS correlated in the non-clinical sample, but because neither variable correlated with children’s self-reported anxiety or children’s worry here, multiple regression was performed instead. In addition to parents’ POS, we found significant correlations in the non-clinical sample between children’s NRT and parents’ NEG, which were included in the regression model as covariates. Due to violated assumptions of normally distributed residuals and heteroskedastic residuals, robust regression was performed via bootstrapping with 10,000 iterations. As described above, the MI boot approach [63] was used to pool confidence intervals. Children’s POS were predicted significantly via children’s NRT, parents’ POS and parents’ NEG in all 20 imputed datasets with *p* < .05 (Δ*F*_pooled_(3, 33) = 4.351, 95%-CI_pooled_ [3.197; 5.505]). Overall, the variables explained together 28% of the variance in POS (*R*^2^_pooled_ = 0.285, corrected *R*^2^_pooled_ = 0.219). Parents’ POS (*B* = 0.434, 95%-CI_pooled_ [0.104; 0.906]) turned out to be an independent predictor of children’s POS. Neither children’s NRT (*B* = 0.027, 95%-CI_pooled_ [-0.014; 0.105]) nor parents’ NEG (*B* = 0.168, 95%-CI_pooled_ [-0.143; 0.370]) predicted children’s POS significantly above the other variables.

## Discussion

We aimed to replicate results of Esbjørn et al. [33] regarding relationships between parents’ metacognitive beliefs and children’s anxiety, worry, and metacognitive beliefs. To our knowledge, this is the first study to examine the association between POS and NEG of children with anxiety disorders and their parents specifically. As expected, NEG of children and their parents correlated moderately positively in both samples. Both of our study samples also revealed significant positive associations between children’s anxiety, children’s NRT and children’s worry with both children’s NEG (mostly large effects) and parents’ NEG (moderate effects). We observed an exception in children’s self-reported anxiety symptoms in our clinical sample, that was not related significantly related to parents’ NEG.

Consistent with our hypothesis and results by Esbjørn et al. [33], the results for both samples suggest that children’s NEG fully mediate the relationship between parents’ NEG and children’s worry. Concerning children’s self-reported anxiety symptoms, full mediation was also supported in our clinical and partial mediation in our non-clinical sample. We had no firm hypothesis concerning POS because of their generally ambiguous role. Our clinical sample revealed no significant association between parents’ and children’s POS. This is in line with other studies that studied non-clinical samples [30, 34]. Children’s POS in our non-clinical sample, however, correlated with a large effect with parents’ POS and turned out to be significantly predicted by parents’ POS. This finding is also in accordance with Esbjørn et al. [33], who reported a positive, moderate relationship. Also, parents’ or children’s POS did not correlate with children’s anxiety and worry at all, going along with results by Donovan et al. [30] in case of worry. All these findings support the assumption that NEG play a more important role than POS do in the development and maintenance of anxiety disorders [14].

The highlighted role of NEG is also confirmed by our finding that NEG, but not POS, differed between our clinical and non-clinical samples with medium to large effect. Other researchers have made similar observations in terms of differences in NEG only [15, 16], while others also detected differences in POS [65, 66]. In contrast, we found no group differences in parents’ POS or NEG. To our knowledge, only one study [30] so far examined the difference between metacognitions of parents of children with and parents of children without anxiety disorders. In line with our result, they did not identify any significant group differences in parents’ POS or NEG.

In addition to potential direct metacognitive transmission, we also exploratively investigated whether parents’ anxiety, worry and parenting stress were associated with children’s metacognitive beliefs, because they are known to be relevant in anxiety transmission research [17, 19, 27, 29, 30].

**Table 2:** Intercorrelations for outcome variables for clinical and non-clinical samples

Non-clinical sample	1	2	3	4	5	6	7	8	9	10	11	12	13
Clinical sample	Age_child_^a^	Sex_child_^a^	ANZ_child_	ANZ_parent_	MKF-K POS	MKF-K NEG	EWC-C	PTQ	BSI-A/P	ESF	MKF-30 POS	MKF-30 NEG	EWC-A
1. Age_child_^a^		.156	− .253^B^	− .213	− .098	− .088^B^	− .063^B^	.131^B^	− .251^B^	− .124	− .335^*,B^	− .252^B^	− .239^B^
2. Sex_child_^a^	.326^**^		.131	− .095	.017	− .047	.254	.198	− .385^*,B^	− .225^B^	− .079	− .199	− .059
3. ANZ_child_	.098^ A^	− .040		.410^**^	.201	.563^***^	.756^***^	.647^***^	.243	.266^*^	.052	.441^**^	.228
4. ANZ_parent_	.022	− .086	.398^***^		.103	.296^*^	.238	.217	.516^***,B^	.594^***,B^	.063^B^	.507^***^	.329^*^
5. MKF-K POS	− .221	− .183	.209^*^	.249^*^		.132	.218	.278^*^	.021	.048	.539^***,B^	.295^*^	.088
6. MKF-K NEG	.351^**,A^	.122	.573^**^	.228^*^	.130		.466^**^	.575^***^	.161	.197	.252	.381^**^	.135
7. EWC-C	.235^ A^	.131	.663^***^	.185	.120	.501^***^		.804^***,B^	.156	.147	.109	.345^*^	.130
8. PTQ	.407^**,A^	.098	.685^***^	.196	.071	.702^***^	.644^***,A^		.111	.128	.249	.366^**^	.132
9. BSI-A/P	.211^ A^	.207^ A^	.183	.238^*,A^	.199	.241^*^	.191	.203		.291^*^	.295^*,B^	.519^***^	.461^**^
10. ESF	.016	.033^ A^	− .040	.283^*,A^	.044	− .053	>.001	.045	.099		− .288^*,B^	.390^**^	.093^B^
11. MKF-30 POS	.284^*,A^	− .097	.194	.311^**,A^	.176^ A^	.277^*^	.228^*^	.429^***^	.507^***,A^	.274^*,A^		.224^B^	.396^**^
12. MKF-30 NEG	.228^ A^	− .097	.155	.338^**^	.111	.242^*^	.247^*^	.328^**^	.569^***^	.126	.682^***,A^		.358^*^
13. EWC-A	.121^ A^	.066	.169	.268^*^	.252^*^	.131	.299^**^	.230^*^	.328^**^	.325^**,A^	.480^***^	.442^***^	

*Note.* Clinical sample: *n* = 68, non−clinical sample: *n* = 40. Pearson’s product−moment correlations for the clinical sample are shown below the diagonal and Pearson’s product−moment correlations for the non−clinical sample are shown above the diagonal. ANZ = Questionnaires for anxiety− and obsessive−compulsive−disorders of DISYPS−II, child = assessment of child/adolescent, parent = assessment of parent, EWC−C = Measure of excessive worry content – child version, PTQ = Perseverative Thinking Questionnaire, MKF−K= Metacognitions Questionnaire for children, POS = positive beliefs about worry, NEG = negative beliefs about worry, BSI−A/P = Brief Symptom Inventory, sum score of Anxiousness and Phobic fear scales, ESF = Parenting stress questionnaire, EWC−A = Measure of excessive worry content – adult version, MKF−30 = Metacognitions Questionnaire – short version

^a^_two−tailed test. *p < .05, ** *p* < .01; ***p < .001_. ^A/B^_Correlation differed significantly between clinical and non−clinical sample_

Parents’ anxiety symptoms in our clinical sample correlated moderately with children’s NEG, and parents’ worry correlated moderately with children’s POS. Neither parents’ worry nor parents’ anxiety symptoms did correlate significantly with children’s POS or NEG in the non-clinical sample. Associations between parents’ anxiety symptoms and worry and children’s metacognitions have, to our knowledge, been investigated in two studies with non-clinical samples to date. They differ from our results in some aspects: Lønfeldt [31] reported children’s POS and NEG both to be related to parents’ anxiety symptoms, while we only found a correlation with NEG exclusively in our clinical sample. Unlike our observation of parents’ worry correlating with children’s POS in our clinical sample, Donovan et al. [30] found a significant association with children’s NEG. Whether parenting stress plays a role in children’s metacognitions also warrants further investigation. We could not detect significant correlations between parenting stress and children’s metacognitions in one of our samples.

Overall, our results support a possible metacognitive transgenerational transmission effect. Parents’ NEG and children’s anxiety symptoms and worry correlate and this relationship is mediated by children’s NEG. This indicates that metacognitions might be transmitted by parents to their children, which then in turn would affect the emergence of excessive worry and pathological anxiety according to the MCM [12–14]. With regard to the role of POS, we cannot draw any clear conclusions as we only detected transgenerational intercorrelations in our non-clinical sample and an association between children’s POS and parents’ worry in our clinical sample. Children’s POS might thus be influenced somehow by parents’ POS and parents’ worry. It remains questionable whether this potential transmission effect influences the frequency and intensity of worries and the development of pathological anxiety. In contrast, our results indicate that parents’ NEG, as well as their anxiety symptoms might affect the development and maintenance of children’s NEG and, subsequently, children’s pathological worry and anxiety.

However, no causal conclusions can be drawn, and replication of our results is needed. Further research on the transgenerational metacognitive transmission should focus on specific metacognitions such as POS and NEG in order to better understand their individual role in this process. Another potentially relevant research topic would be to investigate how exactly such metacognitive transmission might manifest. Esbjørn et al. [33] have hypothesized that parents transmit verbal information about worry to their children, which lead to the child forming corresponding metacognitions. Donovan et al. [30] emphasize that “parents may inadvertently model, prompt, and reinforce worry and its accompanying cognitive factors” (p. 2). This would require examining whether and how parents talk to their children about worry or demonstrate worry processes to their children, and how that affects children’s POS and NEG. Whether known factors in transmitting anxiety such as parenting stress or parenting behavior also influence the development of POS and NEG in children should be investigated further in this context. It would be of particular interest to find out whether a certain communication and parenting style has a preventive effect on the formation of NEG in particular, as the current state of research supports their highlighted role as ascribed in the MCM (compare [11]).

### Limitations

Our study has some limitations that should be considered when interpreting our results. First, our study relies on cross-sectional data. Our correlative findings therefore, enable no causal conclusions about hypothesized transgenerational transmission mechanisms. Second, as we included children with different primary anxiety disorders and possible comorbidities in different treatment settings, our clinical sample is heterogeneous. Unfortunately, we cannot make any statements about the anxiety diagnoses and comorbid diagnoses that the clinicians actually made, as such data was not retrievable in our study’s context. We also have no information on whether participating parents are now undergoing or have ever undergone treatment for mental health problems. Third, we had missing data, which we suspect was because children filled in their paper-pencil questionnaires at home. To deal with missing data, we proceeded according to the current state-of-the-art procedure via Multiple Imputation [61, 67]. Fourth, the internal consistency of the scales assessing NEG and POS in parents and NEG in children was below the acceptable range and below the original validation studies [46], so those results should be interpreted with caution.

### Summary

Anxiety disorders are the most prevalent of mental disorders, affecting approximately one in ten children or adolescents. There is ample evidence that transgenerational transmission plays a central role especially regarding anxiety disorders. Current state of research also shows that metacognitive beliefs are related to anxiety and worry in children and adolescents. As little research has addressed how such metacognitions emerge in children and adolescents to date, we investigated whether transgenerational transmission is also evident in conjunction with POS and NEG. We included *N* = 111 parent-child-dyads, thereof *n* = 71 8- to 16-year-olds with anxiety disorders and *n* = 40 non-clinical controls of the same age group. We demonstrated that children’s metacognitions at least partially mediate the association between parents’ metacognition and children’s anxiety and worry for NEG in both samples. Children’s NEG differed between the clinical and non-clinical sample. The results for POS were more heterogeneous as we only found POS of parents and their children to be related in the non-clinical sample. Overall, these results suggest that NEG may be transgenerationally transmitted, and that this might play a role in how children’s pathological anxiety and worry develop. Our results do not enable us to confirm an influence by potential transgenerational transmission of POS on the development and maintenance of anxiety disorders in children and adolescents. Since we cannot make any causal assumptions based on our cross-sectional data, further research and longitudinal data are needed.

## Data Availability

The data presented in this study is stored at the University of Marburg and can be accessed on request there. The data is not publicly available due to the fact that this is not in accordance with consent provided by participants on the use of confidential data.
